# Influence of dietary protein and fructooligosaccharides on fecal fermentative end-products, fecal bacterial populations and apparent total tract digestibility in dogs

**DOI:** 10.1186/s12917-018-1436-x

**Published:** 2018-03-20

**Authors:** Carlo Pinna, Carla Giuditta Vecchiato, Carmen Bolduan, Monica Grandi, Claudio Stefanelli, Wilhelm Windisch, Giuliano Zaghini, Giacomo Biagi

**Affiliations:** 1 Department of Veterinary Medical Sciences, University of Bologna, via Tolara di Sopra 50, 40064 Ozzano Emilia (Bologna), Italy; 2 Department of Animal Science, Animal Nutrition Unit, Technische Universität München, Liesel-Beckmann-Strasse 2, 85354 Freising, Germany; 3 Department for Life Quality Studies, University of Bologna, Corso d’Augusto 237, 47921 Rimini, Italy

**Keywords:** Dog, Dietary protein, Digestibility, Fructooligosaccharides, Intestinal microbiota, Prebiotics

## Abstract

**Background:**

Feeding dogs with diets rich in protein may favor putrefactive fermentations in the hindgut, negatively affecting the animal’s intestinal environment. Conversely, prebiotics may improve the activity of health-promoting bacteria and prevent bacterial proteolysis in the colon. The aim of this study was to evaluate the effects of dietary supplementation with fructooligosaccharides (FOS) on fecal microbiota and apparent total tract digestibility (ATTD) in dogs fed kibbles differing in protein content. Twelve healthy adult dogs were used in a 4 × 4 replicated Latin Square design to determine the effects of four diets: 1) Low protein diet (LP, crude protein (CP) 229 g/kg dry matter (DM)); 2) High protein diet (HP, CP 304 g/kg DM); 3) Diet 1 + 1.5 g of FOS/kg; 4) Diet 2 + 1.5 g of FOS/kg. The diets contained silica at 5 g/kg as a digestion marker. Differences in protein content were obtained using different amounts of a highly digestible swine greaves meal. Each feeding period lasted 28 d, with a 12 d wash-out in between periods. Fecal samples were collected from dogs at 0, 21 and 28 d of each feeding period. Feces excreted during the last five days of each feeding period were collected and pooled in order to evaluate ATTD.

**Results:**

Higher fecal ammonia concentrations were observed both when dogs received the HP diets (*p* < 0.001) and the supplementation with FOS (*p* < 0.05). The diets containing FOS resulted in greater ATTD of DM, Ca, Mg, Na, Zn, and Fe (*p* < 0.05) while HP diets were characterized by lower crude ash ATTD (*p* < 0.05). Significant interactions were observed between FOS and protein concentration in regards to fecal pH (*p* < 0.05), propionic acid (*p* < 0.05), acetic to propionic acid and acetic + *n*-butyric to propionic acid ratios (*p* < 0.01), bifidobacteria (*p* < 0.05) and ATTD of CP (*p* < 0.05) and Mn (*p* < 0.001).

**Conclusions:**

A relatively moderate increase of dietary protein resulted in higher concentrations of ammonia in canine feces. Fructooligosaccharides displayed beneficial counteracting effects (such as increased bifidobacteria) when supplemented in HP diets, compared to those observed in LP diets and, in general, improved the ATTD of several minerals.

## Background

The bacterial populations that inhabit the intestinal tract play a role of great importance in animal health, as they are involved in nutritional, functional and immunological processes [1]. Several studies have shown that the symbiotic relationship between intestinal microbiota and their host may be positively modulated through dietary strategies that include supplementation with prebiotics and variation of the amount, type and balance of dietary nutrients [2]. Among nutrients, protein represents a key factor in dog nutrition and may have a significant influence on the intestinal microbial fermentations by supporting proteolysis [3]. In particular, even though dogs are a carnivorous species, high amounts of dietary protein have demonstrated to favor a high synthesis of undesirable putrefactive metabolites, such as ammonia and volatile branched-chain fatty acids (BCFA) in the canine hindgut [4, 5].

Several publications have recently highlighted interesting evidence on the use of prebiotic substances both in humans [6] and other species, including companion animals such as dogs [7, 8]. In particular, fructooligosaccharides (FOS) have displayed beneficial effects on the composition [9, 10] and metabolism of canine intestinal microbiota, by favoring a shift from putrefactive to saccharolytic fermentations [11, 12].

Nowadays, the development of nutritional strategies aimed at positively influencing the intestinal health of companion animals is important, with recent trends in canine nutrition focusing on diets rich in proteins (the so-called “ancestral diets”) based on the philosophy of feeding dogs foods similar to those eaten by their wild ancestors [13]. Our hypothesis is that higher dietary protein concentrations may induce an intensification of intestinal putrefactive fermentations and that FOS may counteract this effect. As such, the objective of this study was to evaluate the effects of a dietary supplementation with FOS on fecal bacterial populations, fecal fermentative end-products concentrations and apparent total tract digestibility (ATTD) in dogs fed diets with different protein content.

## Methods

The study was carried out according to the Italian legislation implementing the European Council Directive 2010/63 on the protection of animals used for scientific purposes. The experimental protocol was reviewed and approved by the Scientific Ethics Committee on Animal Experimentation of the University of Bologna. Informed consent was obtained from all dog owners prior to the beginning of the study.

### Animals and diets

Twelve healthy adult dogs (household dogs, different breeds and living in different environments; mean age ± SD = 3.6 ± 1.6) were used. The average body weight ± SD of the dogs was 19.5 ± 6.2 kg. During the study they kept living in their usual environment. Each dog was regularly vaccinated and periodically treated for intestinal parasites; dogs had exhibited no clinical signs of gastrointestinal disorders during the previous 12 months.

Four dry, extruded and complete diets formulated for adult dogs (Effeffe Pet Food S.p.A., Italy), based on cereals, meat and meat by-products, oils and fat, protein plant extract, minerals and yeasts, were used: 1) Low protein diet (LP, crude protein (CP) 229 g/kg dry matter (DM)); 2) High protein diet (HP, CP 304 g/kg DM); 3) LP + FOS; 4) HP + FOS. The sole source of animal protein in the diets was a swine greaves meal (CP 685 g/kg DM; in vitro DM and CP digestibility 0.71 and 0.86, respectively). Fructooligosaccharides (Beneo OPS, FOS from partially hydrolyzed inulin from chicory with a degree of polymerization between 2 and 8; Beneo GmbH, Mannheim, Germany) were incorporated in diets 3 and 4 before extrusion, at a final concentration of 15 g/kg. The experimental diets did not contain significant amounts of soluble fiber sources other than the FOS added to diets 3 and 4. Silica was included in all diets at the dose of 5 g/kg as a source of acid-insoluble ash to be used as a digestion marker. The amount of greaves meal and the presence or not of FOS in the formulation represented the only remarkable difference between the four experimental diets.

In vitro digestibility of the swine greaves meal included in the experimental diets was performed following the method based on the 2-step procedure (2 h incubation with HCl, pepsin and gastric lipase followed by 4 h with pancreatin and bile salts) described by Biagi et al. [14]. Dry ground samples of the swine greaves meal were digested in triplicate. The chemical composition of the experimental diets and the swine greaves meal is shown in Table 1.

**Table 1:** Nutrient analysis (g/kg DM) of the experimental diets and swine greaves meal used as the sole source of animal protein

	Swine greaves meal	LP	HP	LP + FOS	HP + FOS
DM, *as fed*	982	929	935	939	934
CP	685	229	304	241	312
Fat	161	120	140	118	134
Crude ashes	151	66.1	81.6	66.7	81.0
NDF	–	83.0	109	85.0	106
ADF	–	27.0	27.0	28.0	26.0
Starch	–	464	364	456	363
Ca	–	9.15	12.7	10.3	13.9
P	–	6.65	8.05	6.25	7.55
Mg	–	0.80	0.80	0.80	0.80
Na	–	5.70	8.20	5.40	7.45
K	–	7.75	6.05	5.00	6.10
Zn	–	0.23	0.23	0.22	0.23
Mn	–	0.06	0.03	0.05	0.02
Fe	–	0.49	0.39	0.41	0.32
Cu	–	0.02	0.02	0.02	0.02

*FOS* fructooligosaccharides, *HP* high protein diet, *LP* low protein diet

### Experimental design and samples collection

Dogs received four dietary treatments according to a 4 × 4 Latin square experimental design. Each feeding period lasted 28 days, with 12 days wash-out periods in between. During the wash-out periods all dogs were fed the LP diet, as it represented the basal diet during the study. Over the whole crossover study, each dog received all experimental diets, following the same sequence (LP → HP → LP + FOS → HP + FOS) but starting from a different diet.

Dogs were fed twice a day; the daily food amount for each dog was calculated on the basis of the energy content of the experimental diets (calculated using the modified Atwater conversion factors) and the animals’ daily energy requirements, according to the recommendations for the maintenance of small and medium sized adult dogs: 132 kcal/kg BW^0.75^ [15].

On days 0, 21 and 28 of each feeding period a fresh fecal sample was collected from each dog within 30 min from defecation and thereafter frozen at − 80 °C for chemical and microbiological analyses. From days 24 to 28 of each feeding period, feces excreted from dogs were pooled and stored at − 20 °C for nutrients analyses and ATTD assessment.

### Chemical analyses and ATTD calculation

Determination of nutrients in diets, swine greaves meal and fecal samples was performed according to AOAC International standard methods [16] (method 950.46 for water, method 954.01 for CP, method 920.39 for ether extract, method 920.40 for starch, method 942.05 for crude ash). Fiber fractions were determined according to the procedure described by Van Soest et al. [17], where neutral detergent fiber (NDF) was assayed with a heat stable amylase, and acid detergent fiber (ADF) was expressed inclusive of residual ash. Acid-insoluble ash was determined according to Vogtmann et al. [18]. For the determination of minerals, samples of diets and feces were previously diluted with a nitric acid solution (15 M) and processed through microwave mineralization, according to the method US EPA 3052 [19]. The analysis was carried out by inductively coupled plasma-optical emission spectrometry (ICP-OES Optima 2100; PerkinElmer, Waltham, MA, USA). Quantification of macrominerals was performed with the torch in radial position by using a Meinhard nebulizer coupled with a cyclonic spray chamber, while trace elements were assessed with the torch in axial position with the utilization of an ultrasonic wave nebulizer (CETAC U5000; Teledyne Cetac Technologies, Omaha, NE, USA), according to the method US EPA 6010c [20].

Apparent total tract digestibility of DM was calculated using the following equation:

100 − [(100 ×  % marker in the diet)/ % marker in feces]

Apparent total tract digestibility of each nutrient was calculated using the following equation:

100 − [%nutrient in feces × (100 −  % DM digestibility)/ % nutrient in the diet]

Fecal pH was measured using a SevenMulti pH meter (Mettler Toledo, Milan, Italy) on diluted fecal samples (1:10 in distilled water). Ammonia was measured using a commercial kit (Urea/BUN – Color; BioSystems S.A., Barcelona, Spain). Volatile fatty acids (VFA) were analyzed according to Biagi et al. [21]. For the determination of biogenic amines, samples were diluted 1:5 with perchloric acid (0.3 M); biogenic amines were later separated by HPLC and quantified through fluorimetry, according to the method proposed by Stefanelli et al. [22].

### Microbial analyses

Bacterial genomic DNA was extracted and isolated from fecal samples (~ 200 mg) using the QIAamp DNA Stool Mini-Kit (QIAGEN GmbH, Hilden, Germany). Isolated DNA concentration and purity were measured using a NanoDrop 2000c spectrophotometer (Thermo Scientific, Wilmington, DE, USA). Template DNA was diluted to 50 ng/μl and stored at − 20 °C until further analysis. Quantitative Polymerase Chain Reaction (qPCR) was performed using specific primers for total bacteria, *Escherichia coli, Bifidobacterium* genus*, Lactobacillus* genus, *Enterococcus* genus, and *Clostridium perfringens* (Table 2).

**Table 2:** Primers used for quantitative PCR analysis

Target species	Primer	Sequence (5′-3′)	Reference
Total bacteria	FP 16S	GGTAGTCYAYGCMSTAAACG	[53]
	RP 16S	GACARCCATGCASCACCTG	
*Escherichia coli*	*E. coli* F	GTTAATACCTTTGCTCATTGA	[54]
	*E. coli* R	ACCAGGGTATCTAATCC TGTT	
*Bifidobacterium* genus	g-Bifid-F	CTCCTGGAAACGGGTGG	[55]
	g-Bifid-R	GGTGTTCTTCCCGATATCTACA	
*Lactobacillus* genus	Lab-0159	GGAAACAG(A/G)TGCTAATACCG	[56]
	Univ-0515	ATCGTATTACCGCGGCTGCTGGCA	
*Enterococcus* genus	EnteroF	CCCTTATTGTTAGTTGCCATCATT	[57]
	EnteroR	ACTCGTTGTACTTCCCATTGT	
*Clostridium perfringens*	CP1	AAAGATGGCATCATCATTCAAC	[58]
	CP2	TACCGTCATTATCTTCCCCAAA	

Samples were analysed in duplicate in FrameStar® 96 Well Skirted ninety-six-well reaction plates capped with qPCR Adhesive Clear Plate Seal (4titude Limited, Surrey, UK). The qPCR assay was performed using a MasterCycler ep realPlex^4^ (Eppendorf, Wesseling-Berzdorf, Germany). Amplification was performed in duplicate for each bacterial group within each sample. For amplification, 15 μl final volume containing 7.5 μl 2X SensiFAST No-ROX PCR Master Mix (Bioline GmbH, Luckenwalde, Germany), 4.8 μl of nuclease-free water, 0.6 μl of each 10 pmol primer and 1.5 μl of template DNA were used. The amplification cycle was as follows: initial denaturation at 95 °C for 2 min, at 95 °C for 5 s, primer annealing at 55–61 °C for 10 s and at 72 °C for 8 s. The cycle was repeated 40 times. Cycle threshold values were plotted against standard curves for quantification of the target bacterial DNA from fecal samples. To generate standard curves, 10-fold serial dilutions of purified and quantified PCR products were used. The standard curves of the individual qPCR assays were obtained by PCR using specific primers (Table 2) and DNA extracted from the fecal samples. Individual reactions of the standard curves were run in duplicate on each plate for the respective bacterial group. Melting curves were checked after amplification to ensure single product amplification of consistent melting temperature. Results were reported as log10 16S ribosomal DNA gene copies/g fresh matter.

### Statistical analysis

First, for each tested parameter except digestibility data, results obtained after 21 and 28 d of each treatment were compared by one-way ANOVA. Since significant differences were not observed for any of the measured parameters, it was decided to use the mean values obtained from each dog at 21 and 28 d for statistical data analysis.

In the present study a 2 × 2 factorial arrangement of treatments (two different protein concentrations and the presence or absence of FOS in the diet) was used. Data were analyzed by the General Linear Model procedure. The model included dietary protein concentration, FOS and their respective interaction as fixed effects and the dog and period as random effects. Results from samples collected at the beginning (Day 0) of each feeding period were not included in the analysis. Differences were considered statistically significant when *p* < 0.05. All the statistical computations were performed with Statistica 10.0 (Stat Soft Italia, Padua, Italy).

## Results

All the animals remained in good health throughout the study (experimental design provided for a duration of 160 days).

The interaction between protein concentration and FOS influenced fecal pH (*p* < 0.05). Supplementation with FOS resulted in lower pH in feces of dogs receiving the HP diet and, conversely, increased this parameter when dogs were fed with the LP diet (Table 3). Fecal concentrations of ammonia were affected by both dietary protein concentration (41.1 vs. 58.6 μmol/g of feces for LP and HP, respectively; *p* < 0.001) and FOS (53.1 vs. 46.6 μmol/g of feces for diets with or without FOS, respectively; *p* < 0.05) (Table 3). Fecal moisture and concentrations of biogenic amines and total VFA were not affected by treatments (Table 3). A significant interaction between protein concentration and FOS was observed in regards to fecal concentration of propionic acid (*p* < 0.05), acetic to propionic acid ratio (*p* < 0.01) and acetic + *n*-butyric to propionic acid ratio (*p* < 0.01). In particular, FOS decreased propionic acid when dogs were fed with the LP diet and increased it when dogs were fed with the HP diet. Accordingly, in the presence of FOS, the acetic to propionic acid ratio (*p* < 0.01) and the acetic + *n*-butyric to propionic acid ratio were increased in the LP diet and reduced in the HP diet (Table 3).

**Table 3:** Chemical analysis of fecal samples from dogs fed with diets differing in protein concentrations and in presence of fructooligosaccharides

					Anova *p*-value			
	LP	HP	LP + FOS	HP + FOS	Protein concentration	FOS	Protein concentration x FOS	Pooled SEM
pH	6.12	6.58	6.46	6.50	0.008	0.004	0.011	0.07
Water content (mg/g feces)	601	588	615	578	0.354	0.923	0.754	37.6
NH_3_ (μmol/g feces)	39.1	54.1	43.1	63.1	< 0.001	0.027	0.543	4.01
VFA (μmol/g feces)								
Acetic acid	92.8	93.0	85.1	91.3	0.629	0.487	0.649	8.63
Propionic acid	58.3	41.9	44.9	48.7	0.213	0.577	0.049	6.80
*n*-Butyric acid	16.3	19.0	13.9	13.0	0.747	0.136	0.514	2.40
iso-Butyric acid	2.37	2.53	1.80	2.78	0.226	0.652	0.083	0.18
iso-Valeric acid	4.69	4.68	3.30	4.55	0.400	0.327	0.391	0.29
Total VFA	176	159	149	159	0.759	0.286	0.272	10.7
C2:C3	1.62	2.29	2.03	1.97	0.024	0.842	0.008	0.12
C2 + *n-*C4:C3	1.90	2.73	2.35	2.27	0.014	0.831	0.003	0.18
Biogenic amines (μmol/g feces)								
Putrescine	718	765	695	709	0.798	0.668	0.860	90.6
Cadaverine	461	381	348	329	0.641	0.406	0.728	86.5
Spermidine	482	604	491	586	0.129	0.877	0.741	39.4
Spermine	341	271	383	293	0.186	0.495	0.843	49.7

Values are the means of 12 dogs per treatment

*C2/C3* acetic acid/propionic acid ratio, *C2 + n-C4/C3* acetic acid + *n*-butyric acid/propionic acid ratio, *FOS* fructooligosaccharides, *HP* high protein diet, *LP* low protein diet

Most of the bacterial populations evaluated were not modified by any dietary treatment, with the only exception of bifidobacteria for which a significant interaction between protein concentration and FOS was observed (*p* < 0.01). In particular, FOS reduced these bacteria in feces when dogs were fed with the LP diet and, conversely, they increased them with the HP diet (Table 4).

**Table 4:** Microbial analysis (log copies dsDNA/g feces) of fecal samples from dogs fed with diets differing in protein concentrations and in presence of fructooligosaccharides

					ANOVA *p*-value			
	LP	HP	LP + FOS	HP + FOS	Protein concentration	FOS	Protein concentration x FOS	Pooled SEM
Total bacteria	8.87	8.50	8.98	8.87	0.246	0.243	0.543	0.17
*C. perfringens*	5.58	5.44	5.62	5.92	0.623	0.291	0.455	0.23
*Lactobacillus* spp.	8.59	8.19	8.63	8.61	0.250	0.195	0.292	0.14
Enterococci	5.53	5.62	6.00	5.89	0.960	0.139	0.685	0.20
*Bifidobacterium* spp.	5.36	3.92	4.00	4.29	0.032	0.061	0.002	0.21
*E. coli*	5.47	5.12	5.51	5.69	0.699	0.152	0.215	0.17

Values are the means of 12 dogs per treatment

*FOS* fructooligosaccharides, *HP* high protein diet, *LP* low protein diet

Supplementation with FOS significantly improved ATTD of DM (0.89 and 0.85 for diets with or without FOS, respectively; *p* < 0.05), Ca (0.25 and 0.02 for diets with or without FOS, respectively; *p* < 0.05), Mg (0.20 and 0.01 for diets with or without FOS, respectively; *p* < 0.05), Na (0.98 and 0.96 for diets with or without FOS, respectively; *p* < 0.05), Zn (0.34 and 0.19 for diets with or without FOS, respectively; *p* < 0.05) and Fe (0.15 and 0.01 for diets with or without FOS, respectively; *p* < 0.05) (Table 5). High protein diets resulted in lower ATTD of crude ash (0.52 and 0.46 for LP and HP, respectively; *p* < 0.05) (Table 5). Furthermore, a significant interaction between protein concentration and FOS was observed in regards to CP and Mn ATTD (*p* < 0.05 and *p* < 0.001, respectively). In particular, FOS improved the ATTD of both nutrients in the LP diet and decreased it in the HP diet (Table 5). Apparent total tract digestibility of ether extract, P, K, Cu and starch was not influenced by treatments (average ATTD coefficient of starch was 0.99; data not shown).

**Table 5:** Apparent total tract digestibility coefficients in dogs fed with diets differing in protein concentration and in presence of fructooligosaccharides

					ANOVA *p*-value			
	LP	HP	LP + FOS	HP + FOS	Protein concentration	FOS	Protein concentration x FOS	Pooled SEM
DM	0.86	0.85	0.89	0.87	0.136	0.017	0.282	0.01
CP	0.84	0.88	0.90	0.84	0.503	0.528	0.034	0.02
Ether extract	0.97	0.97	0.98	0.97	0.876	0.436	0.397	0.01
Crude ash	0.48	0.47	0.57	0.45	0.024	0.304	0.157	0.03
Macrominerals								
Ca	0.01	0.04	0.25	0.25	0.519	0.010	0.883	0.06
P	0.39	0.40	0.55	0.38	0.353	0.602	0.120	0.04
Mg	0.01	0.01	0.17	0.23	0.607	0.043	0.231	0.08
Na	0.96	0.97	0.97	0.98	0.318	0.034	0.527	0.01
K	0.96	0.95	0.96	0.96	0.224	0.445	0.264	0.01
Trace minerals								
Zn	0.17	0.21	0.36	0.33	0.746	0.042	0.718	0.06
Mn	0.14	0.19	0.92	0.10	< 0.001	< 0.001	< 0.001	0.05
Fe	0.01	0.01	0.18	0.13	0.799	0.025	0.686	0.06
Cu	0.26	0.37	0.45	0.39	0.636	0.166	0.358	0.07

Values are the means of 12 dogs per treatment

*FOS* fructooligosaccharides, *HP* high protein diet, *LP* low protein diet

## Discussion

During the present study, the effects of a moderate FOS supplementation (15 g/kg) on fecal bacterial populations and activity and ATTD were investigated in diets differing in protein content.

Fecal water content was not influenced by dietary treatments and none of the dogs involved in the study showed any gastrointestinal disturbances (such as diarrhea or flatulence). Among the dietary factors causing greater moisture in feces (and negatively influencing fecal quality), there are both the increase of proteolytic fermentations in the hindgut [23] and dietary supplementation with non-digestible oligosaccharides (NDO) [12]. Protein digestion and absorption are considered efficient biological processes in the dog [24]. However, the intake of diets containing large amounts of proteins, even if high digestible, may exceed the digestive/absorptive capacity of the gastrointestinal tract [25]. Consequently, this may lead to a significant increase of proteins reaching the hindgut available for proteolytic fermentations [4, 26], which are known to favor higher osmotic pressure and, consequently, greater water emission into the intestinal lumen [23]. Nevertheless, an increase of fecal water content and negative effects on fecal quality (up to the appearance of diarrhea, in some cases) have been described in dogs receiving diets containing higher protein content compared to that of the HP diets used in the present study (CP 382 and 392 g/kg DM [27]; 655 g/kg DM [28]). It could be presumed that, in the present trial, the HP diets did not affect fecal moisture because of their relatively moderate (and highly digestible) protein content (around 310 g/kg DM).

Moreover, the moderate dose of FOS used in the present study (15 g/kg) might justify the lack of impact on this parameter. In fact, although the physical water-holding properties of NDO and the osmotic action of molecules such as VFA produced by their fermentation are well known [29], several authors, in accordance with the present results, did not report any detrimental effect on fecal moisture (or fecal quality) in dogs when short-chain fructans were supplemented at concentrations lower than 30 g/kg of diet [11, 26, 30].

Interestingly, protein concentration showed a statistically significant, although modest, influence on fecal ammonia, which was increased by HP diets. Ammonia is produced by proteolytic bacteria and represents a toxic and potentially carcinogenic compound that, when present at relevant concentrations in the intestinal lumen, has demonstrated the ability to damage the mucosa [31]. In our study, the increase of fecal ammonia in dogs when fed HP diets induces us to hypothesize increased proteolytic fermentations in the hindgut, as previously suggested [4, 26]. Nevertheless, the fecal concentration of other markers of intestinal bacterial proteolysis (such as BCFAs and biogenic amines) did not increase during HP dietary treatments.

The present results are partially in accordance with recent similar studies in canine species. In a previous similar investigation, the authors described higher fecal ammonia in dogs fed with diets characterized by increasing CP content (from 296 to 485 and 535 g/kg of DM [26]. In a previously cited study, higher ammonia levels (together with higher fecal BCFA concentrations) were observed in feces of dogs fed with a diet containing high amounts of protein (CP from 214 to 655 g/kg of DM) [28]. Similarly, in another study evaluating diets differing in protein content, the authors observed an increase of ammonia and BCFA concentrations when dogs received diets containing higher concentrations of protein (CP from 214 to 392 g/kg of DM) [5].

As described in omnivores such as humans [32] and swines [33], in dogs and cats there is evidence that an increase in dietary protein content increases proteolytic bacteria and reduces microbial populations (in particular, bifidobacteria and lactobacilli) [34, 35] that have been recognized to be beneficial also in these carnivorous species [36]. These outcomes are supported by the well-known “antagonistic pattern” concerning proteolytic bacteria such as clostridia and saccharolytic bacteria such as lactobacilli and bifidobacteria [34]. However, in the present study, protein concentration did not have any effect on the fecal bacterial populations evaluated, partially in accordance with a previously cited study [5]. Conversely, in a previous in vitro trial with canine fecal inoculum, high-protein diets were associated with lower presence of lactobacilli and enterococci [37]. It is possible that in the present study the difference in protein content between LP and HP diets was not large enough to clearly influence fecal microbial populations other than bifidobacteria.

Volatile fatty acids represent the most important fermentative end-products largely produced by bacterial saccharolytic fermentations of non-digestible carbohydrates (such as NDO) [32] and are considered to be beneficial for the host mainly because of their trophic effects on intestinal mucosal cells [38]. For this reason, an increase in VFA production represents a positive outcome often observed during  prebiotics (such as FOS) supplementation, also in canine species [7]. Conversely, FOS (as main effect) failed to exert any positive “prebiotic” outcome on fecal parameters evaluated in the present study. On the contrary, FOS diets increased ammonia concentration in the dogs’ feces. In the previously cited in vitro study with canine fecal inoculum, FOS supplemented in the same LP and HP diets used in the present study (but different in protein digestibility) at the same concentration (15 g/kg), decreased pH values and ammonia and increased VFA levels [37]. However, results from in vivo studies investigating the effects of fructans in dogs are contradictory. In this regard it is well known that ammonia and VFA deriving from microbial metabolism are rapidly absorbed along the intestinal tract and that feces may not reflect their actual concentration in the colon [32, 39].

While these mechanisms could explain the lack of effect of FOS supplementation on fecal VFA observed in the present study, the increasing effect on fecal ammonia is more difficult to explain. According to the present results, other authors reported higher ammonia (as well as iso-valerate and total biogenic amines) fecal levels in dogs receiving fructans at doses between 3 and 9 g/kg [12]. Moreover, an increase of fecal concentration of several proteolytic compounds, including ammonia, BCFA and some biogenic amines has been observed during a study with adult cats receiving a diet supplemented with 40 g of FOS/kg [40]. As suggested by the authors of this latter study, the increase of fecal ammonia observed after a dietary supplementation with FOS may be attributable to a shift of nitrogen excretion from urine to feces, as previously described in other investigations in both dogs [41] and cats [42]. In studies evaluating the effects of FOS in pigs’ diets, a complete FOS fermentation prior to the terminal ileum has been documented [43]. This could favor higher bacterial replication in the small intestine. The absence of carbohydrates and the presence of undigested protein available as a source of energy in the hindgut could have favored increased proteolytic activity by a larger number of bacteria [44]. In canine species, there is a paucity of in vivo studies evaluating the effects of FOS on microbial composition and activity in the ileal digesta (given the obvious ethical limits recently imposed by legislation). A study by Swanson et al. with ileally cannulated dogs reported increased concentration of lactobacilli in ileal digesta (and feces) of dogs fed with diets supplemented with FOS and MOS, supporting the hypothesis that prebiotics are able to exert microbial changes even in the upper intestinal tract [45]. In vitro conditions FOS have shown to be readily fermentable also by canine microbiota [46]. Thus, the higher fecal ammonia observed in present study could be also justified by a potential saccharolytic activity favored by FOS in the small intestine or in the proximal colon that was not maintained throughout the large intestine, due to the depletion of the relatively modest dose of the prebiotic ingested. The greater amount of bacteria reaching the hindgut, associated with the unavailability of carbohydrates, might have led to more intensive putrefactive fermentations [44], as demonstrated by the previously cited investigations in swine species [43].

The significant protein concentration x FOS interactions observed in the present study express a different outcome on some fecal parameters (fecal pH, propionic acid, VFA ratios and bifidobacteria) when dogs consumed FOS supplemented diets in relation to their protein content. In particular, FOS exerted typical prebiotic effects when added to HP diets (as they favored lower fecal pH, higher bifidobacteria and propionic acid concentration and, consequently, lower VFA ratios) and, unexpectedly, they acted in the opposite way in LP diets. Based on the previously described hypotheses concerning the potential early fermentation of FOS before the distal colon, the consequent greater bacterial growth eventually stimulated by this substrate might have favored more intensive proteolytic activity in the hindgut that could justify the effects described for LP diets (higher fecal pH, lower propionic concentration and reduced bifidobacteria). In regards to HP diets outcomes, we can only speculate on the possible influence of FOS and higher amount of proteins along the canine intestinal tract on the potential interactions between microbiota and their metabolites [47] that seem to have partially counteracted the effect observed in LP diets. In this regard, also the decreasing effect of FOS on protein ATTD when dogs were fed with HP diets might represent an expression of the previously described and difficult to explain prebiotic effect. Prebiotics can increase the amount of fecal nitrogen of microbial origin by stimulating microbial growth in the hindgut of dogs [48], with a decreasing effect on protein ATTD [26, 49]. In the present study, FOS exerted this effect only in HP diets presumably because of the higher undigested proteins reaching the hindgut where greater proteolysis might have occurred, with consequent higher fecal nitrogen losses.

The slight improvement of ATTD of DM in diets supplemented with FOS observed in the present study can be presumably attributable to the greater bioavailability of some minerals. In this regard, ATTD coefficients of some minerals (in particular, Ca and Mg) in the diets that were not supplemented with FOS were surprisingly low. During the present study, dogs continued to live with their owners and, for that reason, they drank waters characterized by a potentially different mineral content. Nevertheless, the present investigation was based on a crossover experimental design and so a potential “water effect” on ATTD of minerals was presumably avoided.

Results from the present study seem to confirm that NDO and, in particular, inulin-type fructans like FOS, may improve the intestinal absorption of several macro- and trace minerals, as already observed in previous studies in dogs [8]. Among the mechanisms proposed to explain this effect, the acidification of the intestinal chyme (consequent to an increase in VFA production) may represent the more plausible condition favoring higher solubility and bioavailability of minerals [50]. Previously, some authors reported improved crude ash ATTD (and Mg and Ca ATTD, in particular) in dogs receiving a diet supplemented with oligofructose at 10 g/kg, with no effect on fecal pH [51]. Similarly, in our study, FOS (as main effect) did not reduce fecal pH. However, as previously mentioned, feces may not reflect the status of the intestinal environment and it could be supposed that the greater mineral bioavailability induced by FOS may be the consequence of a temporary acidification of digesta along the ileal and/or colonic tract [52], where FOS might have exerted their best prebiotic effect, as previously speculated.

## Conclusions

Results from the present study show that even a relatively moderate increase of protein in the diet of adult dogs may exert a negative influence on the canine hindgut, as suggested by the increase of fecal ammonia in the dogs when fed with HP diets. Conversely, the supplementation with FOS improved the ATTD of several minerals, suggesting a transitory acidifying effect along the intestinal tract of the dogs. Moreover, this substrate exhibited some opposite outcomes depending on dietary protein content, displaying, in particular, beneficial counteracting effects on a particularly important bacterial population such as bifidobacteria, when added to HP diets.

Certainly, in companion animals more studies are needed to gain a better understanding of dietary effects on gut microbiota and the consequent impact on health.

In fact, at present, the interactions between dogs and cats and their intestinal microbiome are poorly investigated and many assumptions regarding which bacteria are beneficial and which ones may be detrimental derive from human medicine, despite the fact that the optimal composition of the intestinal microbial community may differ between humans and animals with a more carnivorous nature.

## Abbreviations

ADF: Acid detergent fiber
ATTD: Apparent total tract digestibility
BCFA: Branched-chain fatty acids
CP: Crude protein
DM: Dry matter
FOS: Fructooligosaccharides
HP: High protein
LP: Low protein
NDF: Neutral detergent fiber
NDO: Non-digestible oligosaccharides
qPCR: Quantitative Polymerase Chain Reaction
VFA: Volatile fatty acids
